# B-type natriuretic peptides in chronic obstructive pulmonary disease: a systematic review

**DOI:** 10.1186/s12890-016-0345-7

**Published:** 2017-01-10

**Authors:** Nathaniel M. Hawkins, Amit Khosla, Sean A. Virani, John J. V. McMurray, J Mark FitzGerald

**Affiliations:** 1 Division of Cardiology, University of British Columbia, BC Centre for Improved Cardiovascular Health, St. Paul’s Hospital, 1081 Burrard Street, Vancouver, V6Z 1Y6 BC Canada; 2 Glasgow Cardiovascular Research Centre, University of Glasgow, Glasgow, UK; 3 Division of Respiratory Medicine, University of British Columbia and Institute for Heart and Lung Health, Vancouver, Canada

**Keywords:** Natriuretic peptides, Chronic obstructive pulmonary disease, Heart failure, Biomarkers

## Abstract

**Background:**

Patients with chronic obstructive pulmonary disease (COPD) have increased cardiovascular risk. Natriuretic peptides (NP) in other populations are useful in identifying cardiovascular disease, stratifying risk, and guiding therapy.

**Methods:**

We performed a systematic literature review to examine NP in COPD, utilising Medline, EMBASE, and the Cochrane Library.

**Results:**

Fifty one studies were identified. NP levels were lower in stable compared to exacerbation of COPD, and significantly increased with concomitant left ventricular systolic dysfunction or cor pulmonale. Elevation occurred in 16 to 60% of exacerbations and persisted in approximately one half of patients at discharge. Cardiovascular comorbidities were associated with increased levels. Levels consistently correlated with pulmonary artery pressure and left ventricular ejection fraction, but not pulmonary function or oxygen saturation. NP demonstrated high negative predictive values (0.80 to 0.98) to exclude left ventricular dysfunction in both stable and exacerbation of COPD, but relatively low positive predictive values. NP elevation predicted early adverse outcomes, but the association with long term mortality was inconsistent.

**Conclusion:**

NP reflect diverse aspects of the cardiopulmonary continuum which limits utility when applied in isolation. Strategies integrating NP with additional variables, biomarkers and imaging require further investigation.

## Background

COPD is the only major cause of mortality for which death rates continue to rise. There remains a lack of objective measures to risk-stratify patients, standardized management of comorbidities, and therapies that prolong life. One third of deaths in COPD relate to cardiovascular disease, equaling or exceeding pulmonary-related mortality [1–3]. Cardiovascular therapies are proven to reduce morbidity and mortality, yet are underutilized because disease is unrecognized [4]. Simple, generalizable and cost-effective strategies are therefore needed to identify cardiovascular disease (and particularly heart failure) to improve outcomes in COPD.

The U.S. Food and Drug Administration and international guidelines have highlighted the need for biomarker development in COPD [5]. However, development is challenging and translation into clinical practice has been largely unsuccessful [6, 7]. Given the recognized cardiovascular phenotypes within COPD, [8] the use of established cardiovascular biomarkers merits exploration. The natriuretic peptides (NP) B-type natriuretic peptide (BNP) and N-terminal fragment (NT-proBNP) are powerful independent predictors of death and adverse events in HF, a broad range of cardiovascular conditions, and even in asymptomatic individuals in the community [9]. In primary care patients at high cardiovascular risk, intensive management of those with a raised BNP detected on systematic screening reduced the incidence of heart failure and left ventricular dysfunction [10]. NP may therefore prove useful in identifying cardiovascular disease, stratifying risk, and guiding therapy in COPD.

However, pulmonary disease itself, pulmonary hypertension, and right ventricular strain are also associated with NP elevation. This may undermine the utility of NP in COPD across the spectrum of potential applications: reduced diagnostic accuracy for HF; impaired risk stratification due to transient changes or weak association with predictors of prognosis; and by correlation with factors unresponsive to treatment. We therefore undertook a systematic review to direct future research and provide healthcare providers with a concise, critical, unbiased synthesis of the expanding body of literature. The study aims were to define the prevalence, distribution, associations, prognostic implications, and diagnostic accuracy of peptide elevation in COPD.

## Methods

### Participants, outcomes and study designs

Preferred Reporting Items for Systematic reviews and Meta-Analyses (PRISMA) guidelines were followed. The population of interest was patients with COPD receiving natriuretic peptide testing. The outcome of interest was NP, including: levels and proportion elevated in different COPD populations, stratified by COPD severity (stable disease, acute exacerbation (AECOPD), associated cor pulmonale); thresholds used to define abnormal; correlations between NP and measures of ventricular and pulmonary function; risk associated with NP; and accuracy of NP in diagnosing HF. All study designs including cohort, case-control and cross-sectional were accepted.

### Search strategy and data collection

**Fig. 1:**
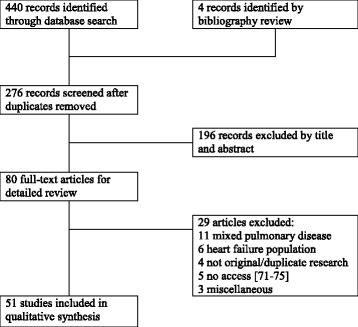
Flow diagram of study selection

MEDLINE (from 1990), EMBASE (from 1990), and the Cochrane Library were searched to June 2015, limited to adult humans, without date or language restriction. Search terms were selected by consensus and iterative database queries. Medical Subject Headings (MeSH) and Emtree terms were identified from keyword mapping and published literature. COPD was identified using MeSH (pulmonary disease, chronic obstructive; bronchitis, chronic), Emtree (chronic obstructive lung disease; chronic bronchitis), and keywords. NP were identified using MeSH (natriuretic peptides), Emtree (brain natriuretic peptide), and keywords. Terms and keywords were combined according to the requirements of the database. The search strategy is outlined in Appendix 1. No review protocol was registered or published. The search identified 440 articles in Medline and EMBASE, totalling 276 records after duplicate removal (Fig. 1). Case reports, reviews and conference abstracts were excluded. Two reviewers (NH and AK) screened titles and abstracts (binary yes/no) with reconciliation through discussion. Studies fulfilling the participant, outcomes and study design criteria were included. Studies involving patients with different pulmonary diseases (as opposed to COPD) or only HF were excluded (Fig. 1). Variables of interest were decided a priori and expanded iteratively after pilot. Excel spreadsheets were employed as data extraction forms and populated directly by both reviewers (NH and AK). The following information was extracted: bibliographic details, sample size and number of centers, population, baseline characteristics and comorbidities, pulmonary function, NP outcomes.

### Study quality

In accordance with the Cochrane Collaboration and Institute of Medicine guidance, risk of bias in observational studies was assessed in selected components with empirical evidence and strong clinical or theoretical grounds. A quality scale was not utilized as many have limited development methodology, validation, arbitrary weightings and inconsistent relationships with effect sizes. 7 bias domains were selected (selection, misclassification, performance, detection, reporting, information and confounding), based on the Cochrane Collaboration Risk of Bias Tool and Handbook and Agency for Healthcare Research and Quality RTI Item Banks,[11–13] Judgement of low, high or unclear risk of bias was assigned for each domain (Appendices 2, 3 and 4).

### Synthesis and analysis

The evidence is presented as a narrative synthesis given the heterogeneous populations, diverse objectives and outcomes examined, varying assays and thresholds, and poorly defined confounding factors. Most importantly, the summary measures presented in many studies (median and ranges) require transformation for meta-analysis. We explored multiple transformation methods, [14–16] all of which declined in accuracy with increasing skew and underestimated the variance by up to half. We identified 4 main groups (stable COPD/BNP, stable COPD/NT-proBNP, exacerbation COPD/BNP, exacerbation COPD/NT-proBNP). Median/IQR was more often reported in the exacerbation and NT-proBNP studies due to skewed distributions (Table 1). Thus transformation for meta-analysis would introduce major error into already large variances in a systematic manner.

**Table 1:** Natriuretic peptides levels in patients with COPD

Stable disease	n	Age Mean ± SD	FEV_1_	FEV_1_ % Pred	Smoking current/past/never	Exacer-bation definition	% LVSD or HF (EF)	Renal function	AF %	NP (pg/ml)	NP levels mean ± SD/SE* or median (IQR)	Controls mean ± SD or median (IQR) *P* value vs COPD	NP levels subgroups mean ± SD or median (IQR)
Fujii [71]	21	68 ± 5	0.94	45	nr	-	ex	normal	nr	BNP	8 ± 2*	-	-
Cabanes [72]	17	65 ± 6	1.3	nr	nr	-	ex	nr	exc	BNP	14 ± 12	-	-
Hemlin [73]	25	66 ± 1	0.8	34	28/72/0	-	ex	normal	exc	BNP	21 ± 5*	-	-
Papaioannou [74]	49	66 ± 9	nr	42	49/nr/nr	-	ex	nr	exc	BNP	31 (15–70)	-	-
Kim [75]	22	73 ± 6	nr	46	nr	-	nr	nr	nr	BNP	41 ± 60	-	-
Anderson [17]	93	68 ± 2	nr	70	34/66/0	-	1 (<40%)	nr	nr	BNP	29 ± 6*	26 (20–32) *p* = 0.46	-
Gemici [18]	17	53 ± 11	nr	55	nr	-	ex	normal	nr	BNP	21 ± 16	13 ± 11 *p* > 0.05	-
Rutten [24]	200	73 ± 5	nr	84	nr	-	15 (≤45%)	nr	9	BNP	39 (17–79)	-	LVSD 135 (41–317), *p* < 0.001
Rutten [24]	200	73 ± 5	nr	84	nr	-	15 (≤45%)	nr	9	NT–BNP	117 (72–210)	-	LVSD 560 (169–1572), *p* < 0.001
Watz [30]	170	64 ± 7	nr	56	42/nr/nr	-	3 (≤50%)	nr	nr	NT–BNP	67 (40–117)	-	-
Murphy [76]	25	66 ± 9	0.95	40	88/12/0	-	12 (<55%)	exc renal failure	nr	NT–BNP	113 (147)	-	LVSD 296, *p* = 0.01
Gale [25]	140	67 ± 13	1.2	nr	82/11/6	-	11 (<45%)	Cr mean 92 μmol/l	9	NT–BNP	44 ± 132	-	LVSD 537 (119–2243), *p* = 0.03
Macchia [26]	218	70 ± 70	1.25	39	24/72/4	-	14 (≤40%)	5% renal failure	nr	NT–BNP	103 (49–273)	-	LVD 677 (384–1682), *p* < 0.0001
Patel [40]	118	68 ± 9	1.22	49	36/nr/nr	-	nr	nr	nr	NT–BNP	12 (6–21)	-	
Boschetto [21]	23	69 ± 4	nr	78	nr	-	ex	eGFR mean 66	nr	NT–BNP	121 (59–227)	50 (43–51) *p* = ns	-
Wang [22]	80	70 ± 6	nr	nr	nr	-	ex	eGFR mean 73	nr	NT–BNP	245 (196–336)	101 (56–150)	-
Rubinsztajn [77]	81	65 ± 7	nr	52	nr	-	nr	nr	nr	NT–BNP	190 ± 234	-	-
Sanchez [78]	71	65 ± 7	nr	39	10/90/0	-	ex	nr	exc	NT–BNP	79 ± 70	-	-
Beghe [23]	70	69 ± 8	nr	60	nr	-	ex	nr	nr	NT–BNP	115 (50–364)	50 (43–51) *p* < 0.05	-
Ozdemirel [19]	31	61 ± 8	1.60	57	39/55/6	-	ex	exc renal failure	exc	NT–BNP	100 ± 82	48 (35) *p* = 0.003	
Bando [27]	14	75 ± 1	1.09	57	nr	-	nr	exc renal failure	nr	BNP	13 ± 3*	7 ± 1	CP 81 ± 13, *p* < 0.001
Bozkanat [28]	38	59 ± 7	nr	40	nr	-	ex	nr	nr	BNP	21 ± 10	9 ± 3	CP 74 ± 36, *p* < 0.001
Anar [29]	80	nr	nr	32	nr	-	nr	exc renal failure	nr	NT–BNP	58 ± 64	-	CP 869 ± 1135, *p* < 0.001
Coldea [79]	72	59 ± 7	1.8	nr	69/nr/nr	-	ex	eGFR median 57	nr	NT–BNP	204 (69–311)	-	CP 1323 (234–2567), *p* < 0.001
Exacerbation													
Xie [80]	174	72 ± 6	nr	47	nr	Hospital	nr	nr	nr	BNP	254 (100–521)	7 (5–10)	-
Escande [81]	29	66 ± 10	nr	37	27/nr/nr	Hospital	ex	eGFR median 92	exc	BNP	37 (21–78)	-	-
Gariani [47]	57	76 ± 8	nr	nr	nr	Hospital	23 (<50%)	nr	28	BNP	420 ± 426	-	-
Abroug [46]	148	68 [15]	nr	nr	nr	ICU	18 (<50%)	Cr med 93 μmol/l	nr	NT–BNP	398 (673)	-	HF 5374 (8243), *p* < 0.0001
Martins [82]	149	77 ± 11	nr	nr	nr	Hospital	51 HF	17% renal failure	37	NT–BNP	268 (482)	-	-
Marteles [83]	99	74 ± 8	nr	nr	nr	Hospital	ex	exc renal failure	nr	NT–BNP	1289 ± 1875	-	-
Chang [44]	244	72 ± 11	0.81	35	33/63/3	Hospital	ex	9% renal failure	nr	NT–BNP	243 ± 498	-	-
Hoiseth [45]	99	72 ± 9	0.91	33	nr	Hospital	14 HF	Cr med 65 μmol/l	10	NT–BNP	423 (264–909)		HF 1554, *p* = 0.102
Ouanes [43]	120	67 [15]	nr	nr	nr	ICU	17 LVSD	58% renal failure	nr	NT–BNP	3796 ± 5448		LVD 3313 (4603), *p* < 0.001
Akpinar [41]	172	71 ± 10	1.50	56	nr	Hospital	nr	exc renal failure	nr	NT–BNP	1188 ± 3233		
Exacerbation vs Stable Control													
Kanat [31]	30	65 ± 7	nr	67	nr	Hospital	ex	exc renal failure	nr	BNP	405 (184–2108)	101 (63–342) *p* = 0.0001	RVD 1460 (857–3018), *p* = 0.01
Wang [32]	311	75	nr	nr	nr	ED	16 (<45%)	eGFR median 73	9	NT–BNP	840 (248–3334)	208 (187–318)	HF 4828 (2044–9204), *p* < 0.001
Exacerbation vs Stable Phase													
Stolz [33]	208	70 ± 10	0.93	41	45/47/8	ED	10	8% renal failure	nr	BNP	65 (34–189)	45 (25–85) *p* < 0.001	CM 144 (58–269), *p* < 0.001
Inoue [35]	60	nr	nr	nr	nr	Mixed	6 (<50%)	nr	nr	BNP	80 ± 16*	41 ± 9 *p* = 0.004	
Nishimura [36]	61	75 ± 8	nr	81	nr	Hospital	6 (<50%)	nr	nr	BNP	55 (27–129)	18 (10–45) *p* < 0.0001	
Lee [37]	18	71	0.8	36	nr	Hospital	28 LVSD	exc renal failure	nr	NT–BNP	630 (220–2500)	147 (7–980) *p* = 0.04	
Patel [38]	98	72 ± 8	1.14	52	20/nr/nr	Antibiotics ± steroids	nr	nr	nr	NT–BNP	36 ± 57	23 ± 39 *p* < 0.001	
El Mallawany [39]	20	58 ± 9	nr	nr	nr/nr/25	ICU	20 LVSD	nr	nr	NT–BNP	1298 ± 849	539 ± 485 *p* = 0.03	HF: 6777 ± 1434

*AF* atrial fibrillation; *BNP* brain natriuretic peptide; *CM* cardiomyopathy; *Cr* creatinine; *eGFR* estimated glomerular filtration rate (mL/min/1.73 m^2^); *exc* excluded; *ICU* intensive care unit; *IHD*, ischaemic heart disease; *LVD* left ventricular dysfunction; *LVSD* left ventricular systolic dysfunction; nr not reported; *NT*-*proBNP* N-terminal proBNP; *RVD* right ventricular dysfunction

## Results

Fifty one studies were identified, of which 31 were published within the preceding 5 years and 46 within the last decade.

### Study quality

Risk of bias in many domains was low with respect to measurement of NP. Studies were typically small, prospective, without interventions or exposures, cohort or cross-sectional in design, and measured NP in all patients using commercial validated assays. However, approximately 50% of studies exhibited selection bias, 20% lacked objective definition of COPD, and 40% failed to report sufficient information to facilitate interpretation of NP levels (e.g. presence of HF) (Appendices 3 and 4).

### Natriuretic peptides levels in patients with COPD

#### Stable COPD

BNP and NT-proBNP levels were normal or only mildly elevated in stable ambulatory patients in whom HF was excluded or infrequent (Table 1). In the seven studies with controls, NP levels were mildly elevated (albeit significantly) in two studies and similar to controls in the remainder [17–23]. The three largest prospective cohort studies in stable COPD included a higher proportion of patients with left ventricular systolic dysfunction (LVSD) (prevalence 11 to 15%) [24–26]. In these patients, NP were elevated approximately 5 fold compared to those without LVSD. Natriuretic peptides were also significantly elevated in patients with cor pulmonale according to various definitions [27–29].

Eight studies examined NP in stable patients stratified by severity of COPD according to the Global Initiative for Chronic Obstructive Lung Disease (GOLD) (Appendix 5). The 5 largest studies observed no significant difference in median or mean levels with severity, while the 3 smallest studies reported significantly higher NP levels in patients with more severe COPD. A single study in 170 patients reported the proportion of patients with elevated BNP stratified by COPD severity [30]. NT-proBNP was elevated in GOLD stages I to IV in 21, 21, 23 and 28% of patients, respectively (*p* = 0.87).

#### Acute exacerbation COPD

Average natriuretic peptide levels were modestly higher during exacerbations than in stable patients in three types of comparison (Table 1): relative to reported values from other studies in stable COPD, compared to stable controls recruited in the same study, [31, 32] and compared to repeated estimates in the same patient outside of an exacerbation episode [33–39]. The time course of biomarker release relative to exacerbation was rarely investigated. In 127 consecutive hospitalizations, NT-proBNP was elevated in 60% of patients at admission and persisted in 28% at discharge [34]. The largest study with multiple time points found no significant decline in average NT-proBNP sampled on days 3, 7, 14 and 35 after the occurrence of exacerbation [38]. Of interest, significant elevation in NT-proBNP in that study were limited to patients with a history of ischaemic heart disease.

#### Subgroups with comorbidities

In subgroups of patients with comorbidities associated with NP release, levels were significantly increased compared to those without comorbidities. These included ischaemic heart disease, [38, 40] pulmonary emboli, [41] arrhythmia, [32] aortic stenosis, [25] pulmonary hypertension, [42] renal impairment [32, 43]. However, these comorbidities were rarely reported or searched for systematically. For example, atrial fibrillation was only reported in 7 studies.

### Correlates and predictors of elevated natriuretic peptides in COPD

The most consistent association was between NP and pulmonary artery pressure, with correlation coefficients ranging from 0.28 to 0.68, typically being around 0.5 (Table 2). In most studies with echocardiography, NP elevation was associated with left ventricular ejection fraction (LVEF) among patients with stable and exacerbation of COPD, [25, 26, 32, 35, 37, 39] even in the absence of raised pulmonary artery pressures. Right ventricular function was rarely characterized, and then using a variety of measures including ejection fraction,[19] tricuspid annular plane systolic excursion (TAPSE), [37] right ventricular diameter and hypokinesia [29, 31]. Heterogeneity and small sample sizes limits interpretation.

**Table 2:** Correlates of natriuretic peptide in patients with COPD

Study	n	Natriuretic peptide	FEV_1_	PaO_2_	Troponin	CRP	LVEF	PAP	RV dysfunction
Echo									
Anar [29]	80 stable	NT–BNP	*r* = −0.06 *p* = 0.73	*r* = −0.14 *p* = 0.40	–	–	*r* = −0.22 *p* = 0.40	*r* = 0.39*p* = 0.01	RVD *r* = 0.36 *p* = 0.02
Bozkanat [28]	38 stable	BNP	*r* = −0.65 *p* < 0.001	*r* = −0.70 *p* < 0.001	–	–	–	*r* = 0.68 *p* < 0.001	–
Chi [84]	61 stable	NT–BNP	*r* = −0.56 *p* < 0.001	*r* = −0.35 *p* = 0.03	–	–	–	*r* = 0.44 *p* = 0.001	–
Hemlin [73]	25 stable	BNP	–	–	–	–	–	*r* = 0.54 *p* = 0.02	–
Hwang [85]	31 stable	NT–BNP	*r* = −0.26 *p* = ns	–	–	–	–	*r* = 0.59 *p* = 0.002	–
Inoue [35]	60 stable	BNP	*p* = ns	*p* = ns	–	–	*r* = −0.41 *p* = 0.02	*r* = 0.5 *p* = 0.004	–
Kim [75]	22 stable	NT–BNP	*p* = ns	–	–	–	–	*r* = 0.51 *p* = 0.02	–
Mansour [86]	57 stable	BNP	*r* = −0.49 *p* < 0.01	*r* = −0.44 *p* < 0.05	–	–	–	*r* = 0.49 *p* < 0.01	–
Ozdemirel [19]	31 Stable	BNP	*r* = −0.44 *p* = 0.001	–	–	–	–	*r* = 0.65 *p* = 0.02	RVEF *r* = 0.09 *p* = 0.51
Kanat [31]	37 AECOPD	BNP	–	*p* = ns	–	–	–	–	*r* = 0.474 *p* = 0.008
Lee [37]	18 AECOPD	NT-BNP	–	–	–	–	*r*_*s*_ = −0.76 *p* < 0.001	p = ns	TAPSE *r*_*s*_ = 0.51 *p* = 0.04
El Mallawany [39]	20 AECOPD	NT–BNP	–	*r* = 0.19 *p* = 0.41	–	*r* = 0.09 *p* = 0.71	*r* = −0.58 *p* = 0.007	–	–
Nishimura [36]	54 AECOPD	BNP	–	–	–	–	*r*_*s*_ = −0.22 *p* = 0.108	–	–
Ouanes [43]	120 AECOPD	NT–BNP	–	–	–	–	*r* = −0.296 *p* = 0.008	–	–
Wang [32]	311 AECOPD	NT–BNP	–	–	–	–	*r* = −0.35 *p* < 0.001	*r* = 0.283 *p* < 0.001	–
No Echo									
Chang [44]	244 AECOPD	NT-BNP	–	*p* = ns	*r*_*s*_ = 0.46 *p* < 0.001	*r*_*s*_ = 0.16 *p* = 0.01	–	–	–
Fujii [71]	21 Stable	BNP	r = −0.30 *p* = ns	r = −0.39 *p* = ns	–	–	–	*r* = 0.28 *p* = ns	–
Hoiseth [45]	99 AECOPD	NT-BNP	–	–	r = 0.34 *p* = 0.0006	–	–	–	–
Martins [82]	173 AECOPD	BNP	–	–	r = 0.06 *p* = 0.4	–	–	–	–
Patel [38]	98 AECOPD	NT–BNP	–	–	*r* = 0.50 *p* < 0.001	*r* = 0.46 *p* < 0.001	–	–	–
Stolz [33]	208 AECOPD	BNP	*r* = 0.104 *p* = 0.222	*r* = 0.115 *p* = 0.191	–	*r* = 0.246 *p* = 0.001	–	–	–

*BNP* brain natriuretic peptide; *FEV*_*1*_ forced expiratory volume in one second; *FVC* forced vital capacity; *GFR* glomerular filtration rate; *IL*–*8* interleukin 8; *LVEF* left ventricular ejection fraction; *NT*-*proBNP* N-terminal proBNP; *PaO*_*2*_ arterial partial pressure of oxygen; *PAP* pulmonary artery pressure; *PVR* pulmonary vascular resistance; *r*_*s*_ Spearman’s rank correlation coefficient; *RV* right ventricle; *RVD* right ventricular diameter; *RVEF* right ventricular ejection fraction; *TAPSE* tricuspid annular plane systolic excursion

The relationship between NP and FEV_1_ or PaO_2_ was inconsistent. Similar to the evidence stratifying by COPD severity, the smaller studies observed significant correlations between NP and both FEV_1_ or PaO_2_. However, correlation coefficients in the two largest studies of 208 and 80 patients were not significant (respectively FEV_1_ *r* = 0.104 and PaO_2_ 0.115; FEV_1_ *r* = 0.06 and PaO_2_ 0.14). A modest significant association was observed between NP and troponin in three studies (*r* = 0.34 to 0.50) [38, 44, 45].

### Prevalence of natriuretic peptide elevation and thresholds employed to define abnormal

Different strategies have been employed to define ‘abnormal’ (Table 3): ROC curve analysis to balance accuracy in predicting specific outcomes; measuring central tendency and dispersion of normal controls (e.g. mean ± 2 SD); manufacturer recommendation; existing publications or investigator selection. The proportion of patients with elevated NP according to these heterogeneous thresholds ranged from 15 to 71% in stable patients, and 16% to 60% during exacerbation. Five studies employed receiver operating curve analysis to determine optimal thresholds for detecting left ventricular dysfunction [24, 32, 39, 43, 46]. However, only one of these studies actually reported the prevalence of an elevated level according to these thresholds (approximately 50% in stable patients) [24]. Moreover, identical thresholds in different studies yielded very different frequencies of elevation. NT-proBNP >125 pg/ml occurred in 23% and 51% of stable patients in two studies [24, 30]. Likewise, NT-proBNP >125 pg/ml occurred in 16%, 27% and 44% of AECOPD in three studies [37, 38, 44].

**Table 3:** Thresholds used to define abnormal in patients with COPD

	Natriuretic peptide	Threshold (pg/ml)	Method of selecting threshold	Proportion elevated (%)
Stable				
Inoue [35]	BNP	34	2 SD from mean of normal control	37
Bozkanat [28]	BNP	36	investigator selection	nr
Rutten [24]	BNP NT–BNP	35 125	ROC curve	49 51
Watz [30]	NT–BNP	125	manufacturer reference range	23
van Gestel [49]	NT–BNP	500	cited review article (Jelic 2006) [87]	17
Macchia [26]	NT–BNP	160	median	nr
Andersen [42]	NT–BNP	95	ROC for echo pulmonary hypertension	71
Anar [29]	NT–BNP	125/450 (age specific)	manufacturer reference range	15
Rubinsztajn [77]	NT–BNP	125	manufacturer reference range	44
Ozdemirel [19]	NT–BNP	84/155 (gender specific)	nr	nr
Exacerbation				
Lee [51]	BNP	88	ROC for survival	39
Gariani [47]	BNP	500	guidelines	30
Abroug [46]	NT–BNP	1000 and 2500	ROC rule out and in LV dysfunction	nr
Sanchez-Marteles [88]	NT–BNP	500	ROC for survival	53
Chang [44]	NT–BNP	220 pmol/l	local laboratory (also Lee 13) [37]	27
Hoiseth [45]	NT–BNP	2500	based on Abroug [46]	18
Marcun [34]	NT–BNP	–	age/sex adjusted 95 percentile	60
Ouanes [43]	NT–BNP	1000/2000 (renal specific)	ROC for LV dysfunction	nr
Lee [37]	NT–BNP	220 pmol/l	local laboratory (also Chang 11) [44]	44
Wang [32]	NT–BNP	935	ROC for LV dysfunction	nr
Patel [38]	NT–BNP	220 pmol/l	based on Chang [44]	16
El Mallawany [39]	NT–BNP	900	ROC for LV dysfunction	nr

*BNP* B-type natriuretic peptide; *COPD* chronic obstructive pulmonary disease; *LV* left ventricular; *NT*-*proBNP* N-terminal proBNP; *ROC* receiver operator characteristic; *SD* standard deviation

### Accuracy of natriuretic peptides in detecting heart failure in patients with COPD

Natriuretic peptides were always significantly elevated in patients with COPD and concurrent HF or LVSD compared to those without (Table 1). However, very few studies examined predictive accuracy to identify HF or LVSD, with just a single study in patients with stable COPD (Table 4) [24]. Four natriuretic peptide assays produced comparable results in 200 stable elderly patients with a clinical diagnosis of COPD. Each test excluded HF with reasonable accuracy (all negative predictive values above 0.85, with positive predictive values approximately 0.4). In three studies of patients with AECOPD, NP demonstrated high negative predictive values (0.80 to 0.98) to exclude left ventricular dysfunction applying thresholds exceeding the manufacturers’ guidance (Table 4) [32, 46, 47]. However, as in the stable population the positive predictive values were relatively low. Two studies also assessed ability to detect systolic and diastolic dysfunction separately [24, 47]. The receiver operating characteristic areas and overall accuracy in the latter were lower though remained acceptable.

**Table 4:** Accuracy of natriuretic peptides in predicting left ventricular systolic dysfunction

	*n*	Population	%LVSD (LVEF)	Threshold	Left ventricular dysfunction	NPV	PPV
Rutten [24]	200	primary care elderly	15 (≤45%)	BNP 35 pg/ml NT-BNP 125 pg/ml	panel adjudicated systolic dysfunction	~0.95	~0.4
Abroug [46]	148	intensive care unit	18 (<50%)	NT-BNP 1000 pg/ml	panel adjudicated systolic or diastolic dysfunction	0.94	0.78
Gariani [47]	57	hospitalization retrospective	23 (<50%)	BNP 500 pg/ml	systolic dysfunction diastolic dysfunction	0.88 0.80	0.47 0.41
Wang [32]	311	hospitalization	16 (<45%)	NT–BNP 935 pg/ml	panel adjudicated systolic or diastolic dysfunction	0.98	0.47

*BNP* B-type natriuretic peptide; *LVEF* left ventricular ejection fraction; *LVSD* left ventricular systolic dysfunction; *NPV* negative predictive value; *NT*-*proBNP* N-terminal proBNP; *PPV* positive predictive value

### Prognostic significance of natriuretic peptides in COPD

We identified 12 studies (6 stable and 6 exacerbation of COPD) reporting the association between NP and prognosis, in which the prognostic significance of elevation was inconsistent (Table 5). Among stable patients, the association between NP and survival over 1 to 4 years failed to remain significant after multivariable adjustment in 3 studies [25, 35, 48]. However, NT-proBNP >500 pg/ml predicted one year mortality in 144 patients with predominantly mild to moderate COPD and preserved LVEF (>40%) undergoing major vascular surgery (adjusted HR 7.7 [95% 1.6–37.4]) [49]. NT-proBNP was also associated with all-cause mortality in a larger cohort of 220 elderly men with COPD (adjusted HR 1.61 [1.27–2.06]), although 26% of that cohort had documented HF [50].

**Table 5:** Prognostic significance of natriuretic peptides in COPD

	n	Follow up	Echo (%)	Heart failure details	Natriuretic peptide threshold	Endpoints	Unadjusted risk	Adjusted risk
Stable								
Inoue [35]	60	3 years	53	6% <50%	BNP > 34.2	death exacerbation	not significant increased	not significant HR 3.8 (1.2–12.7) *p* = 0.02
Gale [25]	140	1 year	100	11% EF < 45%	highest vs lowest quartile	death hospitalization	RR 3.0 (p = 0.001)	not significant not significant
Waschki [48]	170	48 months	100	–	–	death	HR 1.47 (1.05–2.06)	1.16 (0.97–1.39)
Andersen [42]	117	2.8 years	100	–	NT-proBNP <95 ng/L	death	HR 0.29 (0.09–0.97) *p* = 0.04	–
van Gestel [49]	144	1 year	100	ex EF ≤ 40%	NT-proBNP >500 pg/ml	death	HR 4.5 (1.5–13.5)	HR 7.7 (1.6–37.4)
Zeng [50]	220	22 months	–	26% HF	–	death	–	1.61 (1.27–2.06)
Exacerbation								
Stolz [33]	208	2 year	75	10% LVSD	per 100 pg/ml	death ICU admission	not significant 1.12 (1.03–1.22)	not significant 1.13 (1.0–1.24)
Lee [51]	67	inpatient	–	–	BNP >88 pg/ml	death	–	OR 21.2 (2.5–180.4)
Chang [44]	244	1 year	0	acute cardiac disease ex	NT-proBNP >220 pmol/L	death 30 day death 1 year	OR 9.0 (3.1 – 26.2) *p* < 0.001 1 year not significant	OR 7.5 (1.9–28.9) *p* = 0.004 1 year not significant
Marcun [34]	127	6 month	100	13% EF < 55% 42% DD	age/gender adjusted	death hospitalization	HR 5.49 (1.25-24.00) HR 1.34 (0.84-2.63)	HR 4.20 (1.07-14.01) HR 1.48 (0.60-3.69)
Medina [52]	192	1 year	0	exclude prior	NT-proBNP >588 pg/ml	death	OR 3.90 (1.46-10.47) *p* = 0.006	OR 3.30 (1.11–9.85) *p* = 0.034
Hoiseth [45]	99	median 1.9 years	0	21% vs 9% tertile 3 vs 1	tertile 3 vs 1	death	HR 6.9 (3.0 – 16.0) *p* < 0.0001	HR 3.2 (1.3–8.1) *p* = 0.012

*BNP* B-type natriuretic peptide; *COPD* chronic obstructive pulmonary disease; *DD* diastolic dysfunction; *EF* left ventricular ejection fraction; *HF* heart failure; *HR* hazard ratio; *LVSD* left ventricular systolic dysfunction; *NT*-*proBNP* N-terminal pro BNP; *OR* odds ratio; *RR* relative risk

In patients with AECOPD, NP independently predicted short term outcomes including intensive care unit admission, [33] inpatient and 30 day mortality [44, 51]. Median BNP was also significantly higher in failed (inpatient death or early re-hospitalisation) compared to successful discharges following AECOPD hospitalization (median (IQR) 261 (59–555) vs 49 (24–104) pg/ml) [36]. The relationship with longer term survival was less certain. Natriuretic peptides failed to predict mortality at 1 and 2 years in 244 and 208 consecutive patients hospitalized or presenting to the emergency department with exacerbation [33, 44]. However, elevated NP were independently associated with increased mortality at 6 months, 1 year and nearly 2 years in three subsequent studies (respectively HR 4.2, OR 3.3 and HR 3.2) [34, 45, 52].

## Discussion

### Causes of natriuretic peptide elevation in patients with and without COPD

Myocardial stretch in either ventricle consequent to volume or pressure overload increases NP levels [53]. Causes include heart failure with reduced and preserved ejection, [54, 55] right ventricular failure, [56] pulmonary emboli, [41, 57] acute coronary syndromes, [58, 59] valvular heart disease, [60] and arrhythmias [61]. Advancing age and renal dysfunction are also associated with elevated NT-proBNP concentrations [62]. Many of these factors are present in stable COPD and common non-infective precipitants of exacerbation [32, 41, 63]. The presence and extent of each factor varies significantly from patient to patient, and is largely independent of COPD severity or acute right ventricular dysfunction. Thus NP levels are higher during acute exacerbation or chronic decompensation (cor pulmonale) than stable disease, and exhibit significant variability with skewed distributions.

By systematically searching and aggregating individual studies, our review highlights several new and consistent observations which suggest NP release is multifactorial with limited direct relationship to COPD. First, NP levels are increased even in some patients with mild COPD without arterial hypoxaemia, severe pulmonary hypertension or right ventricular dysfunction. Second, levels are stable or exhibit only a minor gradient with increasing COPD severity. Third, the magnitude of the correlation coefficients (r) suggests only approximately 25% to 50% of the variance (*r* ^2^) in NP is attributable to any single variable. Moreover, correlation between left and right ventricular function is likewise modest (LVEF and TAPSE *r* = 0.46 in one study), [37] indicating only around 20% of the variance in function of either ventricle is explained by the function of the other.

### Prognostic significance of natriuretic peptides

Individual studies have concluded that NP may be useful in risk stratifying patients with COPD [34, 44, 49]. However, the overall literature has not previously been summarized. The association with longer term outcomes was inconsistent in both stable and exacerbation populations. Our findings highlight many of the challenges in developing biomarker strategies: relatively small sample sizes; variable performance in heterogeneous populations; and failure to replicate findings from derivation to validation cohorts [7]. At present there is insufficient evidence to recommend routine risk stratification using NP.

The more consistent prediction of early outcomes following exacerbations suggests that NP are more strongly associated with acute pathologies rather than COPD itself [33, 44, 51]. The precise causes remains unclear, as risk associated with many acute events improves with time e.g. HF, PE. Nevertheless, unrecognised LVSD undoubtedly underpins many adverse outcomes. While NP levels were typically modest, [44] up to one fifth of patients with AECOPD had marked elevation indicative of probable left heart failure (although acute right ventricular strain remains possible) [45]. Moreover, the significant unadjusted association between NT-proBNP and mortality in one study was nulled after adjustment for LVEF and valvular disease [25]. This hypothesis is further supported by the high prevalence of unrecognised heart failure in imaging and autopsy studies, [64] and the improved outcomes associated with angiotensin converting enzyme inhibitors and beta-blockers in observational COPD studies [65, 66].

### Clinical application of natriuretic peptides in COPD

Natriuretic peptides exhibit lower diagnostic accuracy for HF in COPD than in populations with acute dyspnoea, [67, 68] due to greater overlap of NP distributions in the respective states to be distinguished: levels are elevated in stable and exacerbation of COPD, and lower in stable compared to acute HF. The threshold providing adequate sensitivity and negative predictive value must generate sufficiently few false positives to integrate into systems of care, be cost-effective, and improve outcomes. However, the positive predictive values in the 3 stable or exacerbation populations we identified ranged from 0.4 to 0.47. This compares unfavourably with a recent meta-analysis of NP in the acute care setting, which reported positive predictive values ranging from 0.67 and 0.64 for BNP and NT-proBNP respectively at the guideline recommended lower thresholds, rising to 0.85 and 0.80 respectively for mid-range values [69]. The resulting increase in false positive results will increase demand on imaging services to confirm or refute the diagnosis.

### Directions for future research

To improve generalizability and interpretation, future studies should use validated assays in consecutive patients, and standardized definitions for COPD, HF and comorbidities. Detailed cardiovascular profiles and imaging are needed to systematically define pathologies contributing to NP elevation. Levels should be reported using guideline and manufacturer recommended thresholds, for both the overall population and stratified according to presence or absence of predictors of NP elevation, particularly left ventricular dysfunction. Larger studies examining cause-specific outcomes are needed. Integrating NP with clinical variables and simple investigations such as electrocardiograms should be evaluated to reduce false positive results and develop cost-effective screening strategies. The goal of improving outcomes is particularly challenged by the inconsistent prognostic implications of NP in COPD in studies to date. The greatest incremental prognostic and therapeutic value is likely in populations with unrecognized heart failure and cardiovascular disease amenable to treatment [34, 45, 70].

### Limitations

Most of the identified studies were single centre with limited numbers of patients and endpoints. The patient populations, assays and cutoffs for NP, and definitions of LVSD and HF were heterogeneous. No study systematically defined causes of NP elevation, and the proportion amenable to therapy e.g. arrhythmia, ischaemia, LVSD, pulmonary emboli. These comorbidities will strongly influence every outcome examined, from symptoms to prognosis. The causes of death in relation to NP elevation also require clarification.

## Conclusions

Natriuretic peptides are often increased in patients with COPD, reflecting three complex interwoven aspects of the cardiopulmonary continuum: left heart systolic and diastolic dysfunction; pulmonary vascular and right heart remodelling; and global cardiovascular risk and comorbidities. The additional peptide elevation during exacerbations is likely a marker of both acute strain and varying degrees of underlying cardiopulmonary disease: in some patients effectively a stress test and harbinger of future adverse events. The balance of these pathophysiologic abnormalities within populations is unclear. The goal is to untangle this heterogeneity, to identify individuals at greatest risk and facilitate targeted interventions. Strategies integrating NP with additional variables, biomarkers and imaging require further investigation.

## Figures and Tables

**Fig. 2:**
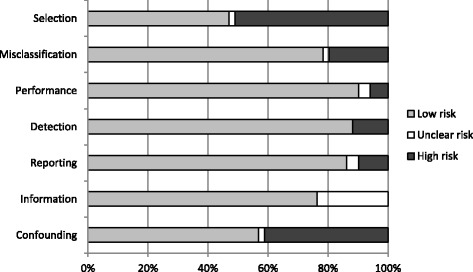
Risk of bias summary

## Appendix 1

Search strategy.

Combined Medline and Embase search strategy.

***Medline and Embase***

1. (exp pulmonary disease, chronic obstructive/or exp bronchitis, chronic/) USE mesd
2. (exp *chronic obstructive lung disease/or *exp chronic bronchitis) USE emezd
3. ((obstruct*) adj2 (pulmonary or lung* or airway* or airflow* or bronch* or respiratory*)).ti,ab.
4. (COPD or COAD or COBD).mp.
5. or/1–4
6. (exp natriuretic peptides) USE mesd
7. (exp brain natriuretic peptide) USE emezd
8. (natriuretic adj2 peptide$ or BNP or proBNP).mp.
9. or/6–8
10. 5 and 9
11. limit 10 to humans
12. limit 11 to yr = ‘1990-Current’
13. 12 not exp newborn/not exp infant/not exp child/not exp adolescent/
14. 13 not (case report* or review* or comment* or editorial* or note* or conference abstract*).pt
15. ..dedup 14

## Appendix 2

**Table 6:** Risk of bias domains assessed

Selection	Is there consecutive or random participant sampling?
Misclassification	Are key inclusion/exclusion criteria clearly stated and defined by valid and reliable measures?
Performance	Did the study vary from the protocol proposed by the investigators, and was there appropriate ethical approval?
Detection	Is the study design prospective, retrospective, or mixed?
Reporting	Are important primary outcomes missing from the results?
Information	Were valid and reliable measures used consistently across all study participants to assess outcomes, exposures or interventions?
Confounding Interpretation	Were important confounding and effect modifying variables accounted for in the design and/or analysis?

Description of 7 risk of bias domains assessed.

## Appendix 3

Risk of bias summary.

Chart demonstrating overall proportion of studies classified as low, unclear or high risk of bias within the 7 domains.

## Appendix 4

**Table 7:** Risk of bias in individual studies

	Selection	Misclassify	Performance	Detection	Reporting	Information	Confounding
Abroug 06 [46]	Low	High	Low	Low	Low	Low	Low
Agoston-Coldea 14 [79]	High	Low	Low	Low	Low	Low	Low
Akpinar 14 [41]	Low	Low	Low	Low	Low	Low	High
Anar 12 [29]	High	Low	High	Low	Low	Low	High
Andersen 12 [42]	High	Low	Low	Low	High	Low	High
Anderson 13 [17]	Low	Low	Low	Low	Low	Low	Low
Bando 99 [27]	High	High	Low	Low	Low	Low	High
Beghe 13 [23]	High	Low	Low	Low	Low	Unclear	High
Boschetto 13 [21]	Low	Low	Low	Low	Low	Unclear	Low
Bozkanat 05 [28]	High	High	Low	Low	Low	Low	Low
Cabanes 01 [72]	Low	Low	Low	Low	Low	Low	Low
Chang 11 [44]	Low	Low	Low	Low	Low	Low	Low
Chi 12 [84]	High	Low	Low	Low	Low	Low	Low
El Mallawany 14 [39]	High	High	Low	Low	Low	Low	High
Escande 14 [81]	High	Low	Low	Low	Low	Unclear	Low
Fujii 99 [71]	High	Low	Low	Low	Low	Low	Low
Gale 11 [25]	Low	Low	Low	Low	Low	Low	Low
Gariani 11 [47]	High	High	Low	High	High	Low	High
Gemici 08 [18]	High	Low	Low	Low	Low	Low	Low
Hemlin 07 [73]	High	Low	Low	Low	Low	Unclear	Low
Hoiseth 12 [45]	Low	Low	Low	Low	Low	Low	Low
Hwang 07 [85]	High	Unclear	Unclear	High	Unclear	Unclear	Unclear
Inoue 09 [35]	High	Low	Low	Low	Low	Low	High
Kanat 07 [31]	Low	Low	Low	Low	Low	Low	Low
Kim 10 [75]	High	Low	Low	Low	Low	Low	High
Lee 04 [51]	High	Low	Unclear	High	High	Unclear	High
Lee 13 [37]	High	Low	Low	Low	Low	Low	Low
Lopez-Sanchez 13 [78]	Low	Low	Low	Low	Low	Unclear	High
Macchia 12 [26]	Low	Low	Low	Low	Low	Low	Low
Marcun 12 [34]	Low	Low	Low	Low	Low	Low	High
Martins 09 [82]	High	High	Low	High	High	Unclear	High
Murphy 09 [76]	Low	Low	Low	Low	Low	Low	Low
Nishimura 14 [36]	Low	Low	Low	Low	Low	Unclear	High
Ouanes 12 [43]	Low	Low	Low	Low	Low	Low	Low
Ozdemirel 14 [19]	Low	Low	Low	Low	Low	Low	Low
Papaioannou 10 [74]	Low	Low	Low	Low	Low	Low	Low
Patel 12 [40]	High	Low	Low	Low	High	Low	High
Patel 13 [38]	High	Low	Low	Low	Low	Low	High
Rubinsztajn 13 [77]	Low	Low	High	High	Low	Low	High
Rutten 07 [24]	Low	High	Low	Low	Low	Low	Low
Sanchez-Marteles 09 [83]	Low	Low	Low	Low	Low	Low	Low
Sanchez-Marteles 10 [88]	Low	Low	Low	Low	Unclear	Low	High
Stolz 08 [33]	Low	Low	Low	Low	Low	Low	High
van Gestel 10 [49]	High	Low	Low	Low	Low	Low	Low
Wang 11 [20]	High	Low	Low	Low	Low	Low	Low
Wang 13 [22]	High	High	Low	Low	Low	Unclear	Low
Wang 13 [32]	Low	High	Low	Low	Low	Low	Low
Waschki 11 [48]	Low	Low	Low	Low	Low	Low	Low
Watz 08 [30]	High	Low	Low	Low	Low	Unclear	Low
Xie 13 [80]	Unclear	High	High	Low	Low	Low	High
Zeng 13 [50]	High	Low	Low	High	Low	Unclear	High

Table reporting risk of bias within the 7 domains for each study.

## Appendix 5

**Table 8:** Natriuretic peptide levels in patients with COPD stratified by severity

	n	Population	Natriuretic peptide (pg/ml)	Median or mean peptide level according to GOLD I/II/III/IV	Significant difference across GOLD groups
Rutten [24]	118	stable	NT-proBNP	~127/119/136/169	*p* = NS
Watz [30]	170	stable	NT-proBNP	69/62/67/73	*p* = 0.78
Inoue [35]	60	stable	BNP	~30/30/50/65	*p* < 0.01
van Gestel [49]	144	stable	NT-proBNP	212/170/352/–	
Mansour [86]	57	stable	BNP	excluded/38/60/78	*p* < 0.05
Chi [84]	61	stable	NT-proBNP	excluded/112/151/250	*p* = 0.02
Nishimura [36]	190	stable	BNP	18/26/22/17	*p* = 0.53
Rubinsztajn [77]	81	stable	NT-proBNP	114/232/155/231	*p* = NS

*BNP* brain natriuretic peptide; *GOLD*, Global Initiative for Chronic Obstructive Lung Disease; *NT*-*proBNP* N-terminal proBNP

Table reporting natriuretic peptide levels in 8 studies stratified by COPD severity according to GOLD classification.

## Abbreviations

AECOPD: Acute exacerbation of chronic obstructive pulmonary disease
BNP: B-type natriuretic peptide
COPD: Chronic obstructive pulmonary disease
FEV_1_: Forced expiratory volume in 1 s
GOLD: Global Initiative for Chronic Obstructive Lung Disease
HF: Heart failure
LVEF: Left ventricular ejection fraction (LVEF)
LVSD: Left ventricular systolic dysfunction
MeSH: Medical Subject Headings
NP: Natriuretic peptides
NT-proBNP: N-terminal pro B-type natriuretic peptide
TAPSE: Tricuspid annular plane systolic excursion
